# Supplementary material for: Experimental Characterization of the Ultrafast, Tunable and Broadband Optical Kerr Nonlinearity in Graphene
Source: Sci Rep. 2019 Jul 22;9:10540. doi: 10.1038/s41598-019-46710-x (PMC6646341; doi:10.1038/s41598-019-46710-x)
Supplement: Supplementary file 1 — Supporting Information - Experimental Characterization of Ultrafast, Tunable and Broadband Optical Kerr Nonlinearity in Graphene [file 41598_2019_46710_MOESM1_ESM.pdf]

SUPPORTING INFORMATION

Experimental Characterization of Ultrafast, Tunable and Broadband Optical Kerr Nonlinearity in Graphene

Siddharatha Thakur,[†,‡] Behrooz Semnani,[†,‡,¶] Safieddin Safavi-Naeini,[†] and Amir Hamed Majedi[∗,†,‡,§]

†*Department of Electrical & Computer Engineering, University of Waterloo, Waterloo, Canada*

‡*Waterloo Institute for Nanotechnolgy, University of Waterloo, Waterloo, Canada*

¶*Institute for Quantum Computing, University of Waterloo, Waterloo, Canada*

§*Department of Physics and Astronomy, University of Waterloo, Waterloo, Canada*

E-mail: ahmajedi@uwaterloo.ca

Phone: +1 519-888-4567 x37443

Theoretical modeling of the nonlinear optical response in graphene

Semiconductor Bloch Equations (SBEs)

It can be shown that the optical response of semiconductor in the continuous excitation regime can be estimated solely based on the dynamics of (i) the microscopic population difference $\mathcal{N}(\mathbf{k},t)$ and (ii) the microscopic polarization $\mathcal{P}(\mathbf{k},t)$. Taking $\mathcal{N}$ and $\mathcal{P}$ as dynamical variables, we obtain the equations of motion for the population difference and the microscopic polarization.

$$\begin{cases} \dfrac{\partial \mathcal{N}(\mathbf{k},t)}{\partial t} - \frac{e}{\hbar}\mathbf{E}.\nabla_{\mathbf{k}}\mathcal{N}(\mathbf{k},t) = -2\Phi(\mathbf{k},t)\mathrm{Im}\left\{\mathcal{P}(\mathbf{k},t)\right\} \\ \\ \dfrac{\partial \mathcal{P}(\mathbf{k},t)}{\partial t} - \frac{e}{\hbar}\mathbf{E}.\nabla_{\mathbf{k}}\mathcal{P}(\mathbf{k},t) = i\varpi_{\mathbf{k}}\mathcal{P}(\mathbf{k},t) + \frac{i}{2}\Phi(\mathbf{k},t)\mathcal{N}(\mathbf{k},t) \end{cases} \tag{1}$$

where $\Phi(\mathbf{k},t)$ is basically the matrix elements of the external potential between the upper and lower energy levels for the given Bloch momentum $\mathbf{k}$ describing direct optical transition.

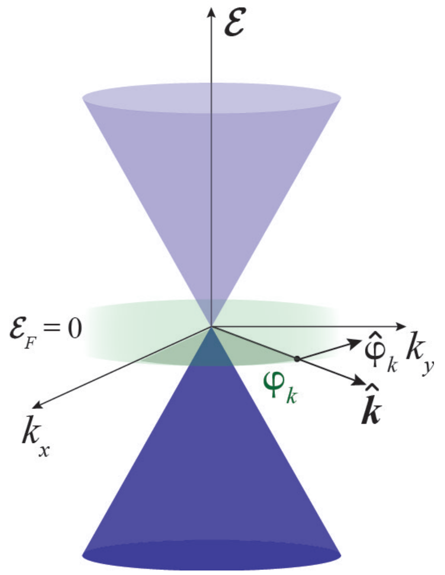

Figure S1: Band structure of graphene showing the excitation pulse with energy $\hbar\omega$, the zero detuning circle $\Delta_k = 0$, and $\hat{\varphi}_k$ is a vector in reciprocal space defined as $\hat{\varphi}_k = \hat{z} \times \mathbf{k}/k$.

Since the momentum of the light is assumed to be negligibly small, we obtain:

$$\Phi(\mathbf{k}, t) = \frac{e\mathbf{E}.\hat{\varphi}_{\mathbf{k}}}{\hbar k} \tag{2}$$

The frequency $\hbar\varpi_{\mathbf{k}} = 2\mathcal{E}_{\mathbf{k}}$ is the energy difference between the upper and lower energy levels and the unit vector $\hat{\varphi}_k$ is defined as $\hat{\varphi}_k = \hat{z} \times \mathbf{k}/k$, shown in Figure S1. At $t = 0$ before the electromagnetic field being applied, the population difference $\mathcal{N}$ is relaxed at its equilibrium $\mathcal{N}_{eq} = f(\hbar v_F k) - f(-\hbar v_F k)$ where f is Fermi-Dirac distribution function. The coupled equations given in (1) are called semiconductor Bloch equations (SBEs) for graphene.

Third Order Frequency Mixing in Graphene

Graphene as a centrosymmetric crystal does not exhibit second order nonlinearity and therefore the first nonlinear term is the third order. We describe the effect of this third order nonlinearity by considering three complex fields with the time dependence of $e^{i\omega_p t}$, $e^{i\omega_q t}$ and $e^{i\omega_r t}$ and their mixing through the third order conductivity of graphene. The third order optical response can be interpreted as a three-photon process and different terms contribute to the third order conductivity tensor namely pure intraband term, pure interband term and combination of the both. There are six distinct photon processes contributing to the third order optics of graphene and are schematically shown in Fig. S2. In compliance with the Boltzmann transport equation, the intraband transitions are displayed by the slight displacement of the Dirac cone in the reciprocal. The interband processes are shown by the vertical transition of the Bloch states- between the upper and lower energy levels-. The adopted mathematical structure of SBEs outlined above would allow us to find the conductivity tensors associated with the six processes shown in Fig. S2.

The nonlinear coefficient associated with each contribution can be compactly expressed

in terms of the the operators $\mathcal{V}_{\mathbf{k}}$ and $\mathcal{W}_{\mathbf{k}}$.

$$\mathcal{V}_{\mathbf{k}}(\omega) = \frac{\gamma_2 + i\omega}{\omega^2 - 2i\gamma_2\omega - \omega_{\mathbf{k}}^2} \frac{e}{\hbar k} \hat{\varphi}_{\mathbf{k}} \tag{3a}$$

$$\mathcal{W}_{\mathbf{k}}(\omega) = \frac{1}{i\omega + \gamma_1} \frac{e}{\hbar k} \hat{\varphi}_{\mathbf{k}} \tag{3b}$$

and the normalized gradient operator $\hat{\mathcal{D}}_{\mathbf{k}}$

$$\hat{\mathcal{D}}_{\mathbf{k}}(\omega) = \frac{1}{i\omega + \Gamma} \frac{e}{\hbar} \nabla_{\mathbf{k}} \tag{3c}$$

where γ_1 and $_2$ are the phenomenological relaxation coefficients associated with the population and polarization (coherence) interband dynamics respectively. Γ is the extrinsic fitting factor accounting for intraband relaxation mechanisms.

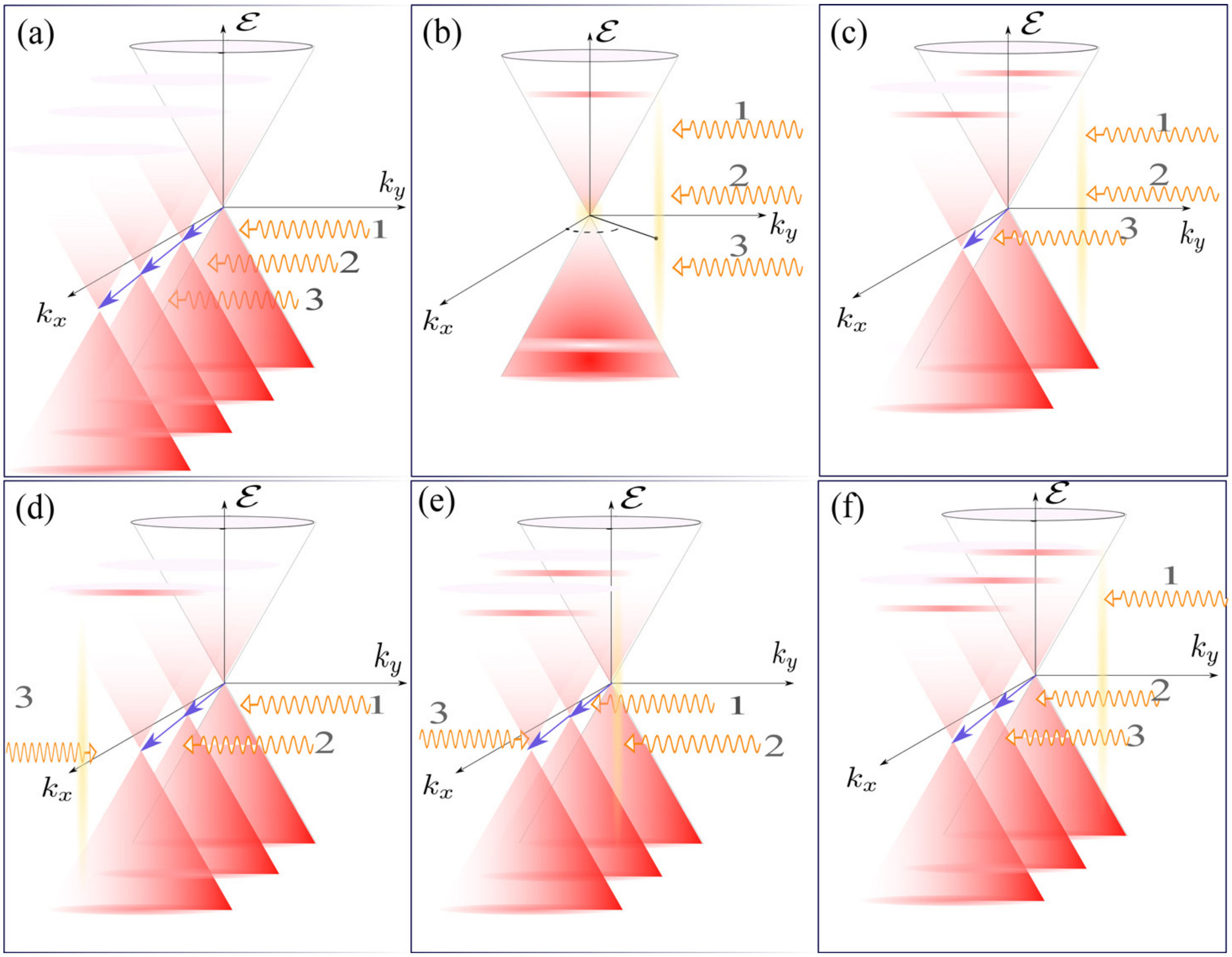

Figure S2: Schematic representation of different three photon processes contributing in the third order nonlinear optics of graphene. The intraband type of dyanamics are displayed by displacement of the distribution and the interband dynamics are shown by two-level transitions. The processes are namely, (a) pure intraband, (b) pure interband, (c) interband-interband-intraband, (d) intraband-intraband-interband, (e) intraband-interband-intraband, and (f) interband-intraband-intraband.[1]

The conductivity tensors for the six distinct photon processes contributing to the third order optical effect in graphene are provided below, with the numeric subscript identifying the type of contribution, i.e. interband, intraband or combination, to the nonlinear response.

[1]Semnani, B., Jago, R., Safavi-Naeini, S., Majedi, A. H., Malic, E. Tassin, P. Anomalous optical saturation of low-energy Dirac states in graphene and its implication for nonlinear optics. 2D Mater. 6, 031003 (2019)

Pure intraband:

$$\overline{\overline{\mathcal{I}}}^{(3)}_{\mathbf{k},1}(\omega_p,\omega_q,\omega_r) = -ev_F\mathcal{P}_I\left\{\ \hat{\mathbf{k}}\hat{\mathcal{D}}_{\mathbf{k}}(\omega_r)\hat{\mathcal{D}}_{\mathbf{k}}(\omega_q)\hat{\mathcal{D}}_{\mathbf{k}}(\omega_p)\mathcal{N}^{eq}_{\mathbf{k}}\ \right\} \tag{4}$$

Pure interband:

$$\overline{\overline{\mathcal{I}}}^{(3)}_{\mathbf{k},2}(\omega_p,\omega_q,\omega_r) = -ev_F\mathcal{P}_I\left\{\hat{\varphi}_{\mathbf{k}}\mathcal{V}_{\mathbf{k}}(\omega_p+\omega_q+\omega_r)\mathcal{W}_{\mathbf{k}}(\omega_p+\omega_q)\mathcal{V}_{\mathbf{k}}(\omega_p)\mathcal{N}^{eq}_{\mathbf{k}}\ \right\} \tag{5}$$

Interband-Interband-Intraband:

$$\overline{\overline{\mathcal{I}}}^{(3)}_{\mathbf{k},3}(\omega_p,\omega_q,\omega_r) = -ev_F\mathcal{P}_I\left\{\hat{\mathbf{k}}\hat{\mathcal{D}}_{\mathbf{k}}(\omega_r)\mathcal{W}_{\mathbf{k}}(\omega_p+\omega_q)\mathcal{V}_{\mathbf{k}}(\omega_p)\mathcal{N}^{eq}_{\mathbf{k}}\ \right\} \tag{6}$$

Intraband-Intraband-Interband:

$$\overline{\overline{\mathcal{I}}}^{(3)}_{\mathbf{k},4}(\omega_p,\omega_q,\omega_r) = -ev_F\mathcal{P}_I\left\{\hat{\varphi}_{\mathbf{k}}\mathcal{V}_{\mathbf{k}}(\omega_p+\omega_q+\omega_r)\hat{\mathcal{D}}_{\mathbf{k}}(\omega_q)\hat{\mathcal{D}}_{\mathbf{k}}(\omega_p)\mathcal{N}^{eq}_{\mathbf{k}}\ \right\} \tag{7}$$

Intraband-Interband-Intraband:

$$\overline{\overline{\mathcal{I}}}^{(3)}_{\mathbf{k},5}(\omega_p,\omega_q,\omega_r) = -ev_F\mathcal{P}_I\left\{\hat{\varphi}_{\mathbf{k}}\hat{\mathcal{D}}_{\mathbf{k}}(\omega_r)\mathcal{V}_{\mathbf{k}}(\omega_p+\omega_q)\hat{\mathcal{D}}_{\mathbf{k}}(\omega_p)\mathcal{N}^{eq}_{\mathbf{k}}\ \right\} \tag{8}$$

Interband-Intraband-Intraband:

$$\overline{\overline{\mathcal{I}}}^{(3)}_{\mathbf{k},6}(\omega_p,\omega_q,\omega_r) = -ev_F\mathcal{P}_I\left\{\hat{\varphi}_{\mathbf{k}}\hat{\mathcal{D}}_{\mathbf{k}}(\omega_r)\hat{\mathcal{D}}_{\mathbf{k}}(\omega_q)\mathcal{V}_{\mathbf{k}}(\omega_p)\mathcal{N}^{eq}_{\mathbf{k}}\ \right\} \tag{9}$$

where $\mathcal{P}_I$ is the intrinsic permutation operator. The overall conductivity tensor is then obtained as

$$\overline{\overline{\sigma}}^{(3)}(\omega_p,\omega_q,\omega_r) = \sum_{l=1}^{6}\sum_{\mathbf{k}}\overline{\overline{\mathcal{I}}}^{(3)}_{\mathbf{k},l} \tag{10}$$

Kerr Coefficient of Graphene

The theoretical prediction of $\sigma^{(3)}_{xxxx}(\omega,\omega,-\omega)$ is plotted in Fig. S3. This part of the nonlinearity contributes in the nonlinear refractive index. This component of the nonlinear response exhibits resonant behavior around $\hbar\omega \sim 2E_f$.

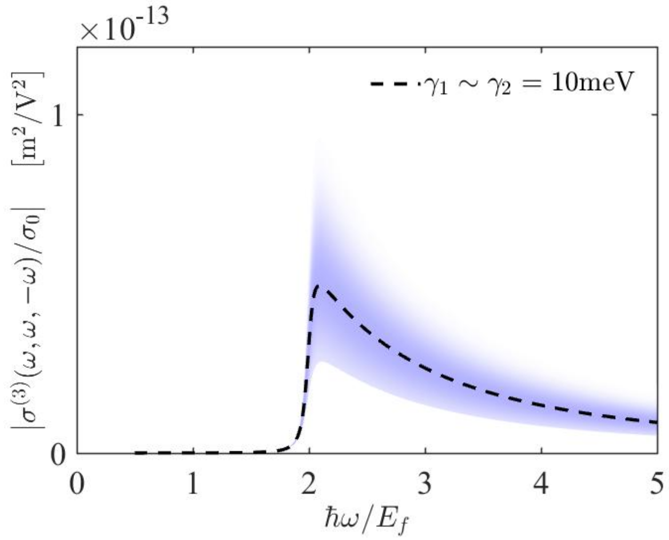

Figure S3: $\sigma^{(3)}(\omega, \omega, -\omega)$ normalized to $\sigma_0 = e^2/4\hbar$ at $T \sim 0$K. Blue shaded regions describe the variations of the coefficients due to the uncertainties in relaxation coefficients.

The equivalent third order bulk susceptibility of graphene is related to the third order surface dynamic conductivity via

$$\chi^{(3)}(\omega_p, \omega_q, \omega_r) = \frac{\sigma^{(3)}_{xxxx}(\omega_p, \omega_q, \omega_r)}{i(\omega_p + \omega_q + \omega_r)d_{gr}\varepsilon_0} \tag{11}$$

where d_{gr} is the equivalent thickness of graphene which is typically around $d \approx 3$ [2] and ε_0 is the free space permittivity. Obviously for the case of graphene, the definition of the nonlinear bulk susceptibility is ambiguous due to the arbitrariness in the definition of the thickness of the two-dimensional structure. In the Kerr-type nonlinear response, the dependence of the complex refractive index n on the intensity of light I is given by

$$n = n_0 + (n_2 - ik_2)I \tag{12}$$

where $I = 2\epsilon_0 \mathrm{Re}\{n_0\}c\,|\mathbf{E}|^2$ (c is the speed of light). The nonlinear coefficient n_2 is related

[2] Semnani, B., Majedi, A. H. Safavi-Naeini, S. Nonlinear quantum optical properties of graphene. J. Opt.18, 035402 (2016)

to the bulk susceptibility $\chi^{(3)}(\omega,\omega,-\omega)$ as[2]

$$n_2 - ik_2 = \frac{3}{4\epsilon_0 c\,|n_0|^2}\chi^{(3)}(\omega,\omega,-\omega)\left[1 - i\frac{\text{Im}\{n_0\}}{\text{Re}\{n_0\}}\right] \tag{13}$$

It is easy to show that for the case of graphene this expression is merely independent of the particular choice of d_{gr} and it introduces an intrinsic parameter. Fig. S4 displays our theoretical prediction for the real and imaginary parts of the Kerr coefficient $n_2 - ik_2$ at the room temperature.

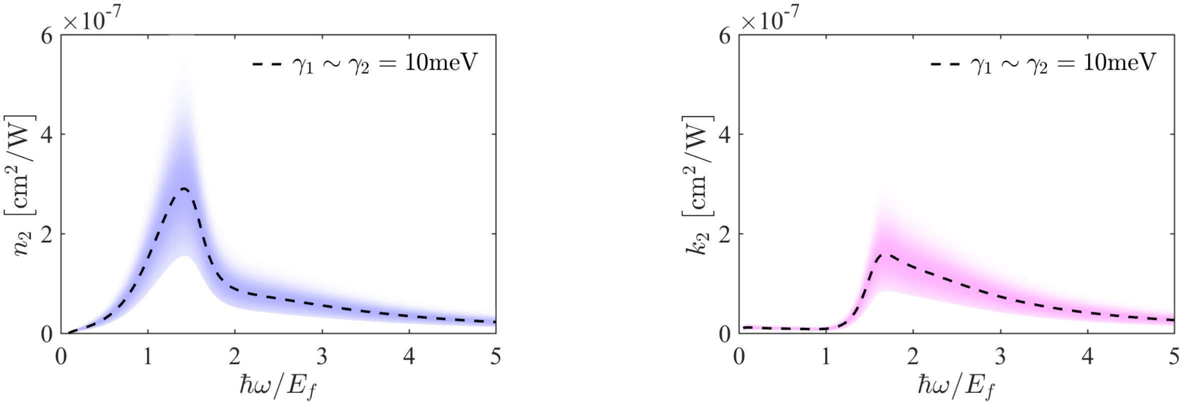

Figure S4: Kerr nonlinear coefficient of graphene [i.e. $n_2 - ik_2$ defined in Eq. (12)] at $T = 300$K. (a) nonlinear refractive index n_2 (b) nonlinear absorption k_2. Over a wide range of frequency the Kerr coefficient is around $\sim 10^{-8}\text{cm}^2\text{W}^{-1}$. Shaded regions describe the variation of the coefficients due to uncertainties in the relaxation coefficients.

Fig. S4 shows that over a wide range of frequency and far from the resonances, the nonlinear coefficient is around $n_2 \sim 10^{-8}\text{cm}^2\text{W}^{-1}$.

Steady-State analysis of the Semiconductor Bloch equations and square wavelength dependency of the Kerr coefficient

The Kerr-trype nonlinearity might have multiple origins including two photon absorption, intraband effects and ultimately optically induced population change. However, the Kerr coefficient over the optical range- where the interband dynamics plays the leading role-,

is mostly originated from the optically induced change into the population difference. In order to capture this effect, we adopt steady state ansatz within the rotating frame approximation [3]. We define $\mathcal{N}(\mathbf{k},t) \approx \widetilde{\mathcal{N}}_{\mathbf{k}}^{st}$ and $\mathcal{P}(\mathbf{k},t) \approx \widetilde{\mathcal{P}}_{\mathbf{k}}^{st} e^{i\omega t}$ where the tilde $\sim$ designates the stationary part of the population difference and polarization with vanishing time derivatives. The steady state solution of the SBEs yields the optically modified population difference and polarization

$$\widetilde{\mathcal{N}}_{\mathbf{k}}^{st} = \mathcal{N}_{\mathbf{k}}^{eq} \frac{\gamma_1}{\gamma_1 + \gamma_2 \left|\mathcal{L}_{\mathbf{k}}(\omega)\right|^2 \left|\Phi_{\mathbf{k}}\right|^2} \tag{14a}$$

$$\widetilde{\mathcal{P}}_{\mathbf{k}}^{st} = \frac{i}{2} \mathcal{L}_{\mathbf{k}}(\omega) \widetilde{\mathcal{N}}_{\mathbf{k}}^{st} \Phi_{\mathbf{k}} \tag{14b}$$

where $\Phi_{\mathbf{k}} = e\mathbf{E}_0.\hat{\varphi}_{\mathbf{k}}/\hbar k$ is a complex phasor associated with $\Phi(\mathbf{k},t)$. Since the steady state solution depends on the field magnitude via $|\Phi_{\mathbf{k}}|^2$ in the denominator of the Eq. (14a), the overall solution is function of the intensity $I_0 = 2\varepsilon_0 c \left|\mathbf{E}_0\right|^2$ (c is the speed of light and ε_0 is the permitivity). The complex Lorentzian $\mathcal{L}_{\mathbf{k}}(\omega)$ is abbreviated as

$$\mathcal{L}_{\mathbf{k}}(\omega) = \frac{1}{\gamma_{\mathbf{k}}^{(2)} + i\Delta_{\mathbf{k}}} \tag{15}$$

Here $\Delta_{\mathbf{k}} = \omega - \varpi_{\mathbf{k}}$ denotes the detuning of the two level system at $\mathbf{k}$ with respect to the excitation.

Due to the ultrafast Rabi oscillations at the zero detuning region (i.e. $\Delta_{\mathbf{k}} \approx 0$) the steady state population difference is modified significantly by the intense illuminating field. One can speculate that the asymptotic frequency dependence of the Kerr coefficient can be gleaned from the steady state inversion around the zero detuning region as

$$\widetilde{\mathcal{N}}_{\mathbf{k}}^{st} = \mathcal{N}_{\mathbf{k}}^{eq} \frac{\gamma_1}{\gamma_1 + \gamma_2 \left|\mathcal{L}_{\mathbf{k}}(\omega)\right|^2 \left|\Phi_{\mathbf{k}}\right|^2}\Bigg|_{\Delta_{\mathbf{k}}\approx 0} \approx \mathcal{N}_{\mathbf{k}}^{eq} \left[1 - \frac{1}{\gamma_1\gamma_2} \left|\Phi_{\mathbf{k}}\right|^2\right] \tag{16}$$

[3] Boyd, R. W., Nonlinear Optics, 3rd ed. Academic Press, (2010)

where $\mathcal{L}_{\mathbf{k}}(\omega) = 1/(\gamma_2 + i\Delta_{\mathbf{k}})$. The corresponding steady state polarization is obtained from Eq. (14b). The induced nonlinear interband current is then calculated as

$$\mathbf{J}_{NL}(\omega) = -\frac{e^2}{\hbar} v_F g_s g_v D \iint_{\text{RBZ}} \mathrm{d}^2\mathbf{k} \left\{ \hat{\varphi}_{\mathbf{k}} (\hat{\varphi}_{\mathbf{k}} \cdot \mathbf{E}) \frac{1}{k} \mathcal{L}_{\mathbf{k}}(\omega) \left[\widetilde{\mathcal{N}}_{\mathbf{k}}^{st} - \mathcal{N}_{\mathbf{k}}^{eq} \right] \right\} \tag{17}$$

$$\sim \beta \frac{e^2}{\hbar} g_s g_v D \frac{1}{\gamma_1 \gamma_2} \left| \frac{e}{\hbar k} \mathbf{E}_0 \right|^2 \mathcal{N}_{\mathbf{k}}^{eq} \bigg|_{k=K_0} \mathbf{E}_0 \tag{18}$$

where $2\hbar v_F K_0 = \hbar\omega$ determines the zero detuning circle around the Dirac point. The dimesionless parameter $\beta \sim \pi$ accounts for the angular integration. Since the population difference $\mathcal{N}_{\mathbf{k}}^{eq}$ is almost constant for $\hbar\omega \gg E_f$, the $1/K_0^2$ term in the above equation dictates the frequency dependence of the Kerr type nonlinearity. Obviously, the nonlinear current is quadratically proportional to the wavelength as $\mathbf{J}_{NL}(\omega) \overset{\propto}{\sim} \omega^{-2} \propto \lambda^2$. It is noted that the quadratic spectral dependence of the Kerr coefficient reflects the peculiar linear dispersion of the quasiparticles.

Negligible Kerr effect in the quartz substrate

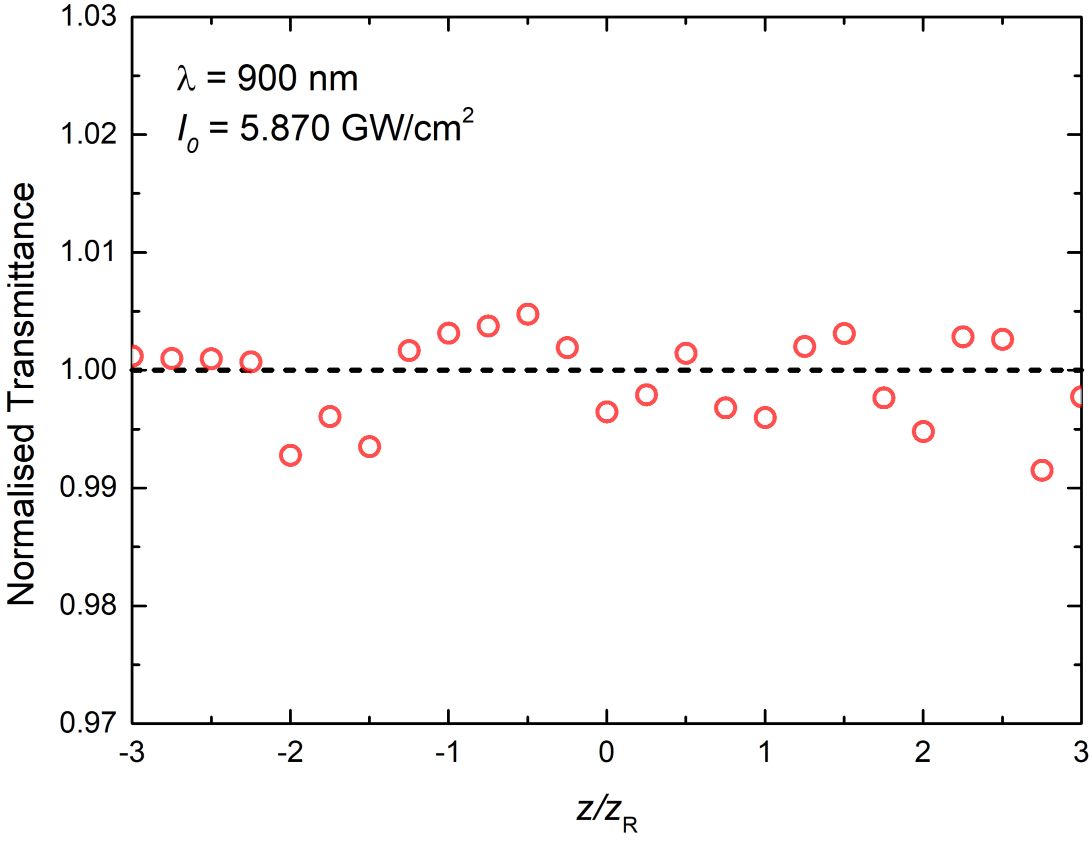

Figure S5: **Z-scan of bare quartz substrate.** Z-scan of bare quartz substrate performed at 900 nm at ~ 6 GW/cm^2 used to verify that the signal obtained in measurements originated from graphene.

Spectral dependence of the effective Kerr coefficient

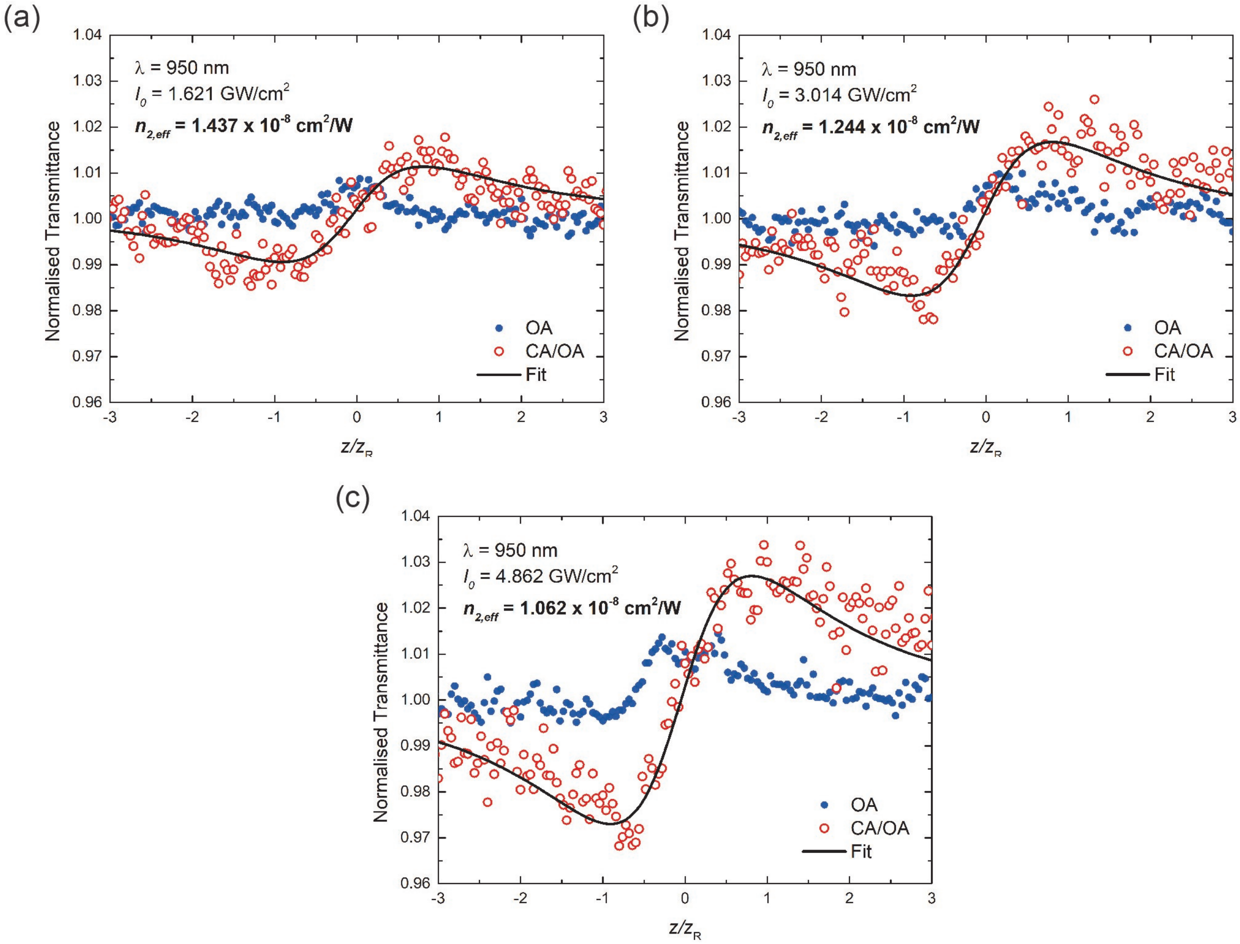

Figure S6: **Irradiance dependence of $n_{2,eff}$ at 950 nm.** Z-scan profiles performed at 950 nm with on-axis irradiances of (a) 1.621 GW/cm^2, (b) 3.014 GW/cm^2, and (c) 4.862 GW/cm^2. The trend shows a decrease in $n_{2,eff}$ with increase in on-axis irradiance, an effect that occurs due to saturation.

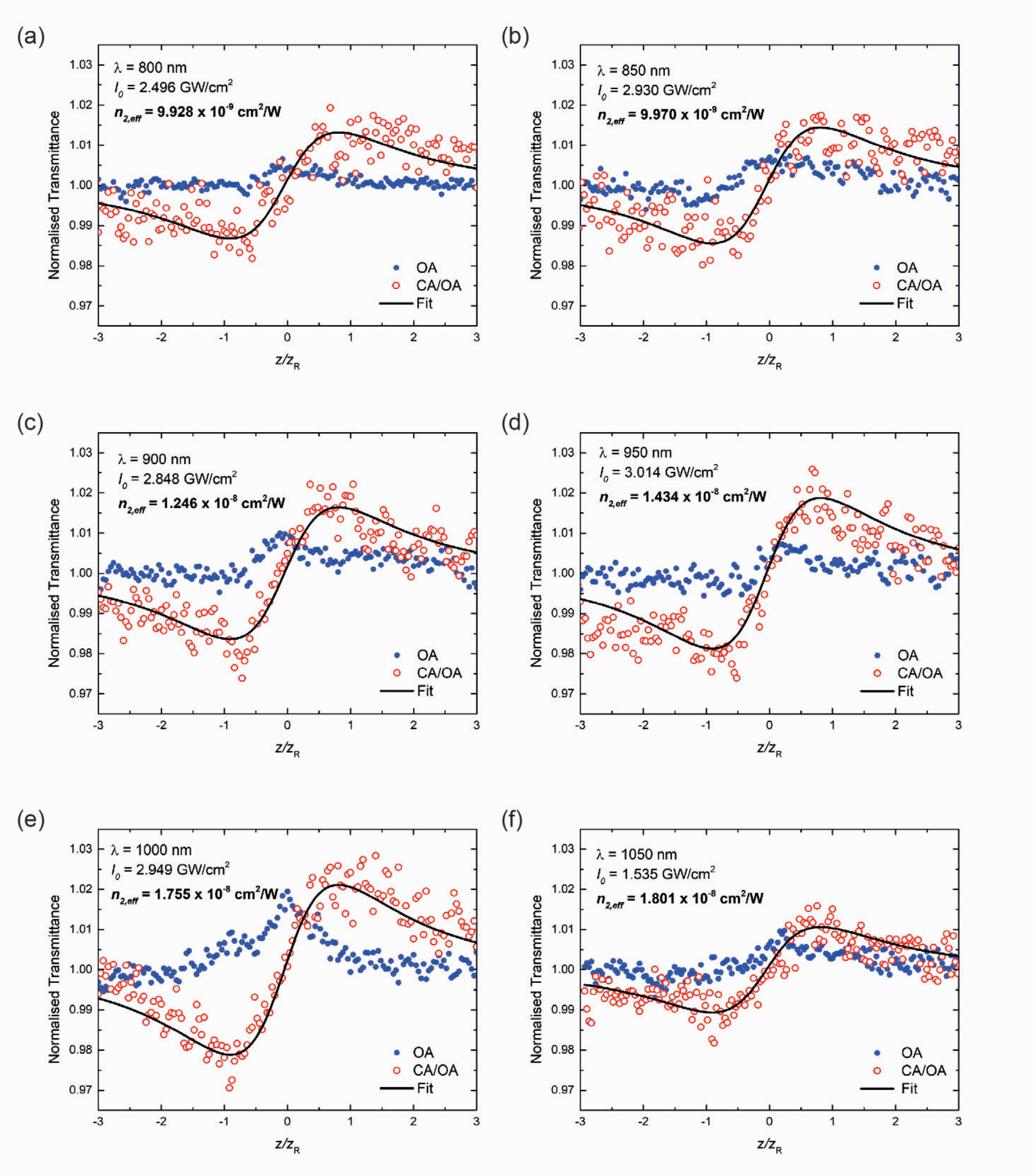

Figure S7: **Z-scan profiles for the spectral-dependence of $n_{2,eff}$ with excitation spanning 800-1050 nm.** Z-scan profiles containing the OA, absorption normalised CA (CA/OA) and the fit. Plots (a-f) show the data and fits for excitation wavelengths of 800, 850, 900, 950, 1000 and 1050 nm, respectively. With the exception of 1050 nm due to source limitation, all data sets are taken at similar on-axis irradiances with the exact values and the corresponding $n_{2,eff}$ values also provided.

Pulse-width dependence of the effective Kerr coefficient

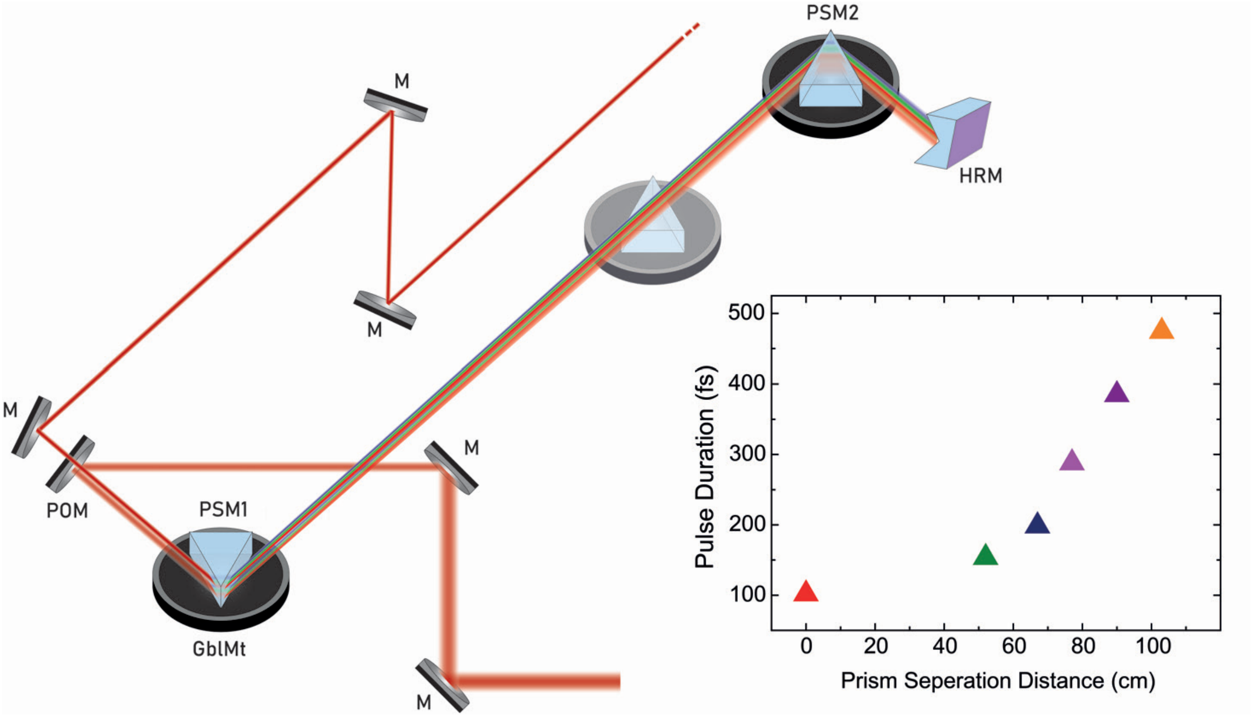

Figure S8: **Dispersion based prism-pair set-up.** The input beam is coupled from laser source and a few mirrors (M) are used to guide the beam into the first prism which is mounted on a gimble mount (GblMt) allowing 6 degrees of freedom. The beam is dispersed by the first prism (PSM1) and the diverging beam is then coupled to a second prism (PSM2) where it collimates on the other side. A hollow roof mirror (HRM) reflects the beam back exactly in the same path, but at a height 1 cm lower than the first beam. The beam follows the same path back and is reflected by a pick-off mirror (POM). PSM2 is moved along the path of the beam to change the separation distance to vary the stretching factor. **(Inset)** The resulting change in pulse duration as a function of the separation distances used in the measurement. The pulse width measurement was performed using a home-built rotating mirror autocorrelator.

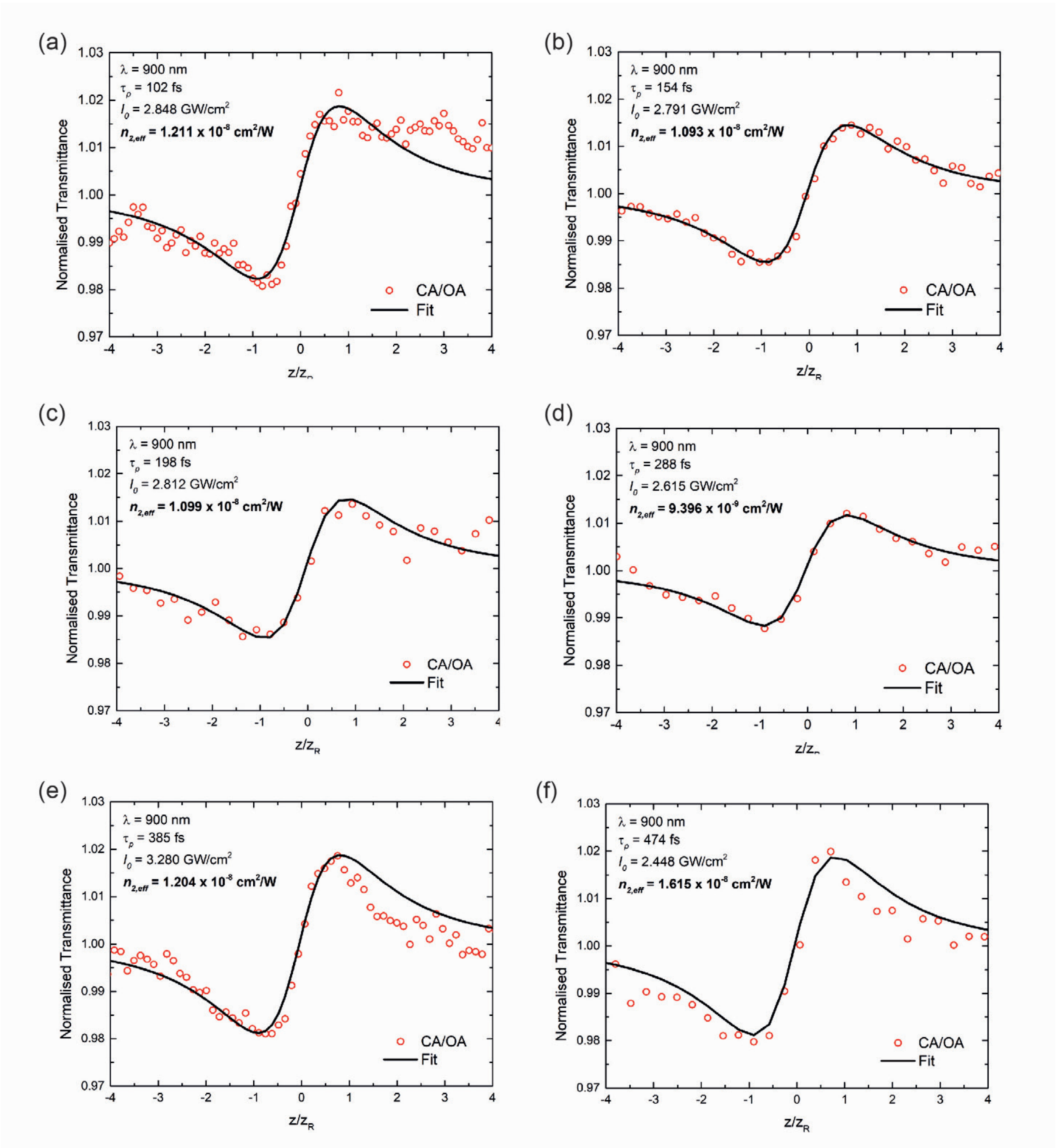

Figure S9: **Z-scan profiles for the pulse-width dependence of $n_{2,eff}$ with pulse duration spanning 100-475 fs.** Z-scan profiles containing the OA normalised CA (CA/OA) and the fit. Plots (a-f) show the data and fits for pulse durations of 102, 154, 198, 288, 385 and 474 fs, respectively.
